# Selective blocking of CXCR2 prevents and reverses atrial fibrillation in spontaneously hypertensive rats

**DOI:** 10.1111/jcmm.15694

**Published:** 2020-08-18

**Authors:** Yun‐Long Zhang, Fei Teng, Xiao Han, Pang‐Bo Li, Xiao Yan, Shu‐Bin Guo, Hui‐Hua Li

**Affiliations:** ^1^ Department of Emergency Medicine Beijing Key Laboratory of Cardiopulmonary Cerebral Resuscitation Beijing Chaoyang Hospital Capital Medical University Beijing China

**Keywords:** atrial fibrillation, chemokine‐receptor, CXCR2, inflammation, oxidative stress, SB225002

## Abstract

Atrial fibrillation (AF) is associated with inflammation and oxidative stress. Recently, we demonstrated that the chemokine‐receptor CXCR2 plays a critical role in the recruitment of monocytes/macrophages and the development of hypertension and cardiac remodelling. However, the role of CXCR2 in the pathogenesis of hypertensive AF remains unclear. AF was induced in Wistar‐Kyoto rats (WKYs) and spontaneously hypertensive rats (SHRs) administered with the CXCR2 inhibitor SB225002. Atrial remodelling, pathological changes and electrophysiology were examined. Our results showed that the chemokine CXCL1 and its receptor CXCR2 were markedly increased in atrial tissue of SHRs compared with WKYs. The administration of SB225002 to SHRs significantly reduced the elevation of blood pressure, AF inducibility and duration, atrial remodelling, recruitment of macrophages, superoxide production and conduction abnormalities compared with vehicle treatment. The administration of SB225002 to SHRs also reversed pre‐existing AF development, atrial remodelling, inflammation and oxidative stress. These effects were associated with the inhibition of multiple signalling pathways, including TGF‐β1/Smad2/3, NF‐κB‐P65, NOX1, NOX2, Kir2.1, Kv1.5 and Cx43. In conclusion, this study provides new evidence that blocking CXCR2 prevents and reverses the development of AF in SHRs, and suggests that CXCR2 may be a potential therapeutic target for hypertensive AF.

## 1 INTRODUCTION

Atrial fibrillation (AF) is the most common type of cardiac arrhythmia and increases the risk for stroke, heart failure and death. However, current antiarrhythmic therapy has moderate efficacy and considerable risks. Thus, there is a need for effective and safe therapeutic drugs for AF. The atrial inflammatory response, oxidative stress and fibrosis are common pathological alterations in AF.^1^ Increasing evidence indicates that inflammation and its related immune response play a crucial role in the initiation and maintenance of AF.^1^, ^2^ Indeed, clinical studies have indicated that the recruitment of immune cells such as monocytes and macrophages and the levels of pro‐inflammatory cytokines, such as C‐reactive protein (CRP), tumour necrosis factor (TNF)‐α, interleukin (IL)‐2, IL‐6, IL‐8 and monocyte chemoattractant protein (MCP)‐1, are significantly increased in the atria of patients with AF.^2^, ^3^, ^4^ An increased inflammatory response has also been observed in angiotensin (Ang) II‐induced mouse AF models.^5^, ^6^ Recent studies have demonstrated that inflammatory pathways contribute to atrial electrical and structural remodelling, thereby leading to increased susceptibility to AF.^1^ For example, nuclear factor‐κB (NF‐κB) is a key transcription factor that mediates the expression of a range of target genes, including IL‐1β, IL‐6, nicotinamide adenine dinucleotide phosphate (NADPH) oxidase 2 (NOX2), NOX4 and connexin 43 (Cx43).^5^ The transforming growth factor‐β (TGF‐β1)/Smad2/3 pro‐fibrotic signalling pathway is critical for atrial fibrosis, a hallmark of AF, that is also mediated by the inflammatory response. Thus, targeting specific inflammatory cascades and mediators may be potential strategies for the prevention and treatment of AF.

Chemokines are a family of chemotactic and pro‐inflammatory cytokines. To date, over 50 chemokines have been identified in humans, which have been classified into four subfamilies, namely CXC, CC, CX3C and XC. These chemokines can bind to approximately 20 chemokine‐receptors, which are respectively grouped into the four subfamilies CXCR, CCR, CX3R and CXR, and are classed as seven transmembrane domain G‐protein‐coupled receptors.^7^ Chemokines and their receptors were originally investigated for their role in the trafficking of leucocytes during inflammatory and immune responses. It is now known that they have important functions under different conditions, such as homoeostasis, tissue repair, tumour growth, inflammatory response and autoimmune diseases.^7^ Among them, CXC subfamily chemokines, such as CXCL1, CXCL2, CXCL3, CXCL5, CXCL7 and CXCL8, can regulate recruitment of monocytes, macrophages and neutrophils in inflammatory diseases by activating their corresponding receptor, CXCR2.^7^ Many studies have demonstrated that CXCL1‐CXCR2 signalling plays a critical role in regulating the trafficking of immune cells and the pathogenesis of several cardiovascular diseases, including myocardial ischaemia/reperfusion injury, myocarditis and atherosclerosis.^8^, ^9^, ^10^, ^11^ Moreover, our recent data indicate that the up‐regulation of CXCL1 or CXCR2 contributes to the Ang II‐induced recruitment of monocytes and macrophages, hypertension, vascular injury and cardiac hypertrophic remodelling. In contrast, genetic ablation or pharmacological blockage of CXCR2 reduces these effects in mice and spontaneously hypertensive rats (SHRs).^12^, ^13^, ^14^ However, whether CXCR2 has a pro‐arrhythmic role and whether blocking CXCR2 could be a new approach for the prevention and treatment of AF remain unknown.

In this study, we examined the impact of the CXCR2 inhibitor SB225002 on the development of AF in SHRs. Our results demonstrated that the levels of CXCL1 and CXCR2 were highly up‐regulated in the atrial tissue of SHRs compared with in WKYs. In contrast, pharmacological inhibition of CXCR2 activity by SB225002 markedly reduced AF inducibility and atrial structural and electrical remodelling in SHRs. These beneficial effects were associated with the inhibition of macrophage infiltration, oxidative stress and multiple signalling pathways (NF‐κB‐P65, NOXs, and TGF‐β1/Smad2/3). Therefore, our results demonstrate that CXCR2 has a crucial function in the development of AF, and suggest that blocking CXCR2 may represent a new therapeutic target for hypertensive AF.

## 2 MATERIALS AND METHODS

### 2.1 Animals and treatment

Two‐month‐old male WKYs and SHRs were obtained from the Jackson Laboratory (Sacramento, CA, USA). The specific CXCR2 inhibitor SB225002 (Selleck, Houston, TX, USA) was administered intraperitoneally (1 mg/kg/d) to the rats from the age of 2 or 6 months and continued for 4 or 5 months, respectively. Blood pressure was measured from the age of 1 month and every month thereafter using a tail‐cuff system (BP‐2010A; Softron, Tokyo, Japan) as described previously.^14^ This study was approved by the Animal Care and Use Committee of Capital Medical University (AEE1‐2016‐045) and conformed to the US National Institutes of Health Guide for the Care and Use of Laboratory Animals.

### 2.2 Induction of AF

The rats were anesthetized with 2.5% tribromoethanol at a dose of 0.02 mL/g (Sigma‐Aldrich, St. Louis, MO, USA), and body temperature was maintained at approximately about 37°C using a mouse pad circuit board (JRD‐7w; Nomoy Pet, Jiaxing, China). Atrial electrophysiology was studied as described previously.^5^, ^6^ Briefly, sinus node recovery time and atrial effective refractory period were analysed according to regular pacing and standard S1S2 pacing protocols.^15^, ^16^ Intracardiac pacing was performed by inserting a Millar 1.9F octapolar electrophysiology catheter (Scisense, London, Ontario, Canada). The atria were paced a 3× threshold and a cycle length of 150 ms or 20 ms shorter than the spontaneous sinus cycle length. Burst pacing containing 200 impulses at 50 Hz was used to induce AF. The duration of the subsequent spontaneous AF after burst pacing was recorded in a computer‐based data acquisition system (GY6328B; HeNan HuaNan Medical Science. & Technology Ltd, Zhengzhou, China). AF was defined as irregular rapid atrial activations with varying electrogram morphology lasting ≥0.5 seconds. Atrial rates were typically >1500 beat/min.

### 2.3 Echocardiographic measurement

Atrial dimension in rats was measured by echocardiography using a Vevo 2100 High‐Resolution Imaging System (Visual Sonics, Inc, Toronto, Ontario, Canada) as reported previously.^14^

### 2.4 Histological analysis

Atrial samples were quickly removed, fixed in 4% paraformaldehyde for 24 hours and embedded in paraffin. Serial sections 5 µm thick were cut and stained with a Masson's trichrome staining kit (Sigma‐Aldrich, St. Louis., MO, USA). Immunohistochemistry was performed with an anti‐Mac‐2 (1:200 dilution; Santa Cruz Biotechnology Inc, Dallas, TX, USA) antibody for 1 hour at room temperature followed by incubation with appropriate secondary antibodies. The areas of fibrosis, α‐SMA‐positive cells and Mac‐2‐positive cells were analysed with NIH ImageJ software (US National Institutes of Health, Bethesda, MD, USA) as described previously.^5^, ^6^ Atrial cryosections at 5 µm ‐thick were stained with dihydroethidine (DHE) at 1 µmol/L in phosphate buffered saline for 30 minutes at 37°C. Fluorescent images were obtained from more than 10 random fields for each sample using a Labophot 2 microscope (Nikon, Tokyo, Japan).^5^, ^6^

### 2.5 Quantitative real‐time PCR (qPCR) analysis

Total RNA was isolated from fresh left atrial tissue using TRIzol (Invitrogen, Carlsbad, CA, USA) according to the manufacturer's protocol. First‐strand cDNA was generated from 1 to 2 μg total RNA by RT Enzyme mix (Accurate Biology, technology Co., Ltd. Hunan, China). The mRNA levels of CXCL1, CXCR2, α‐smooth muscle actin (α‐SMA), collagen I, collagen III, IL‐1β, IL‐6 and TNF‐α were analysed using a PCR thermocycler (S1000 Thermal Cycler; Bio‐Rad, Hercules, CA, USA) as described previously.^14^ The values were normalized to those of glyceraldehyde‐3‐phosphate dehydrogenase (GAPDH). Primer sequences were provided in Table S1.

### 2.6 Immunoblotting analysis

Protein lysates were extracted from left atria, and the concentration was determined using a BCA protein assay. Proteins (40‐60 μg) were subjected to sodium dodecyl sulphate polyacrylamide gel electrophoresis and transferred to a polyvinylidene difluoride membrane (Bio‐Rad), which was incubated with primary antibodies against CXCR2, TGF‐β1, Smad2/3, phosphorylated (p)‐Smad2/3, P65, p‐P65, NOX1 and NOX2 (Cell Signaling Technologies, Boston, MA, USA), and GAPDH (Proteintech Group. Inc, Rosemont, IL, USA).^14^, ^17^ Antimouse or anti‐rabbit IgG secondary antibodies were purchased from Cell Signaling Technologies. All blots were analysed by using the ImageJ and normalized to GAPDH levels.

### 2.7 Statistical analysis

Statistical analysis was performed by using SPSS software version 19.0 (SPSS, Chicago, IL, USA). The normality test (Shapiro‐Wilk) was performed to determine whether the data were normally distributed. The student *t* test was then used to determine the significant difference between two groups in normal distribution. The Mann‐Whitney test was used for the results that were not normally distributed. Repeated‐measures ANOVA was used to analyse the blood pressure data. If the ANOVA demonstrated a significant effect, post hoc comparisons were made pairwise with the Fisher least significant difference test. *P*‐values < 0.05 were considered statistically significant.

## 3 RESULTS

### 3.1 CXCL1 and CXCR2 expression is up‐regulated in the atrial tissue of SHRs

**FIGURE 1:**
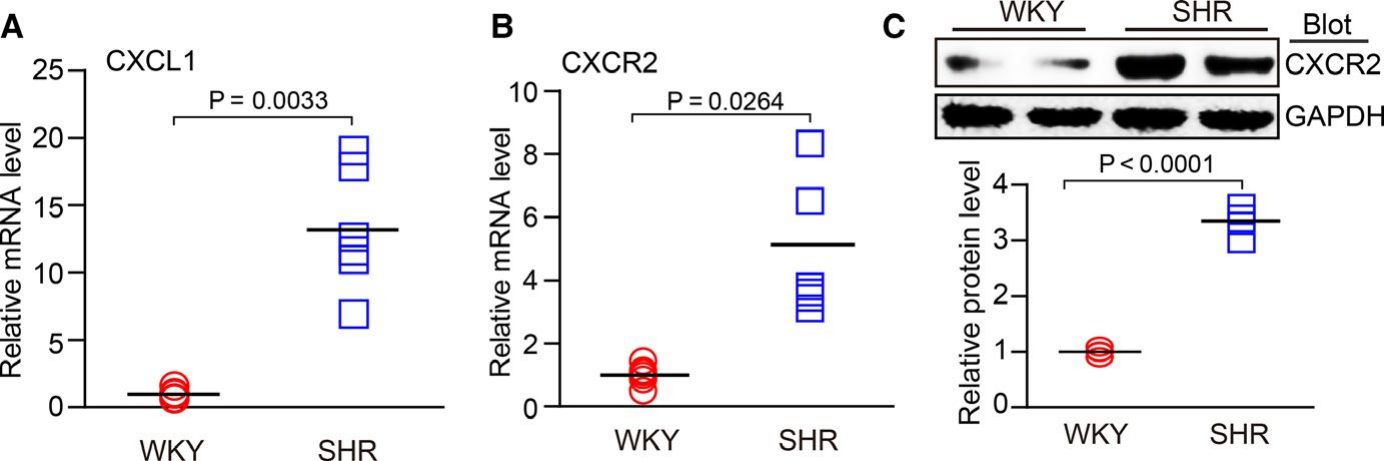
CXCL1 and CXCR2 expression in the atria of WKYs and SHRs. (A, B) qPCR analysis of the mRNA levels of chemokines CXCL1 and CXCR2 in the atria of 6‐month‐old WKYs and SHRs (n = 4). (C) Immunoblotting analysis of CXCR2 protein levels in the atria of 6‐month‐old WKYs and SHRs (upper). Quantification of protein density (lower, n = 4). Results are expressed as the mean ± SEM, and n represents the number of animals in each group

To determine the role of CXCR2 in the regulation of atrial remodelling and AF in SHRs, we first examined the chemokine ligand of CXCR2 (ie CXCL1) in atrial tissue. qPCR analysis revealed that CXCL1 mRNA expression was significantly increased in the atrial tissue of SHRs compared with WKYs at 6 months of age (Figure 1A). Accordingly, CXCR2 expression at the mRNA and protein levels was also markedly higher in the atria of SHRs compared with WKYs (Figure 1B,C). Thus, these results suggest that the up‐regulation of CXCR2 may contribute to the development of AF in SHRs.

### 3.2 Blocking CXCR2 by SB225002 administration reduces AF susceptibility and atrial dilation

**FIGURE 2:**
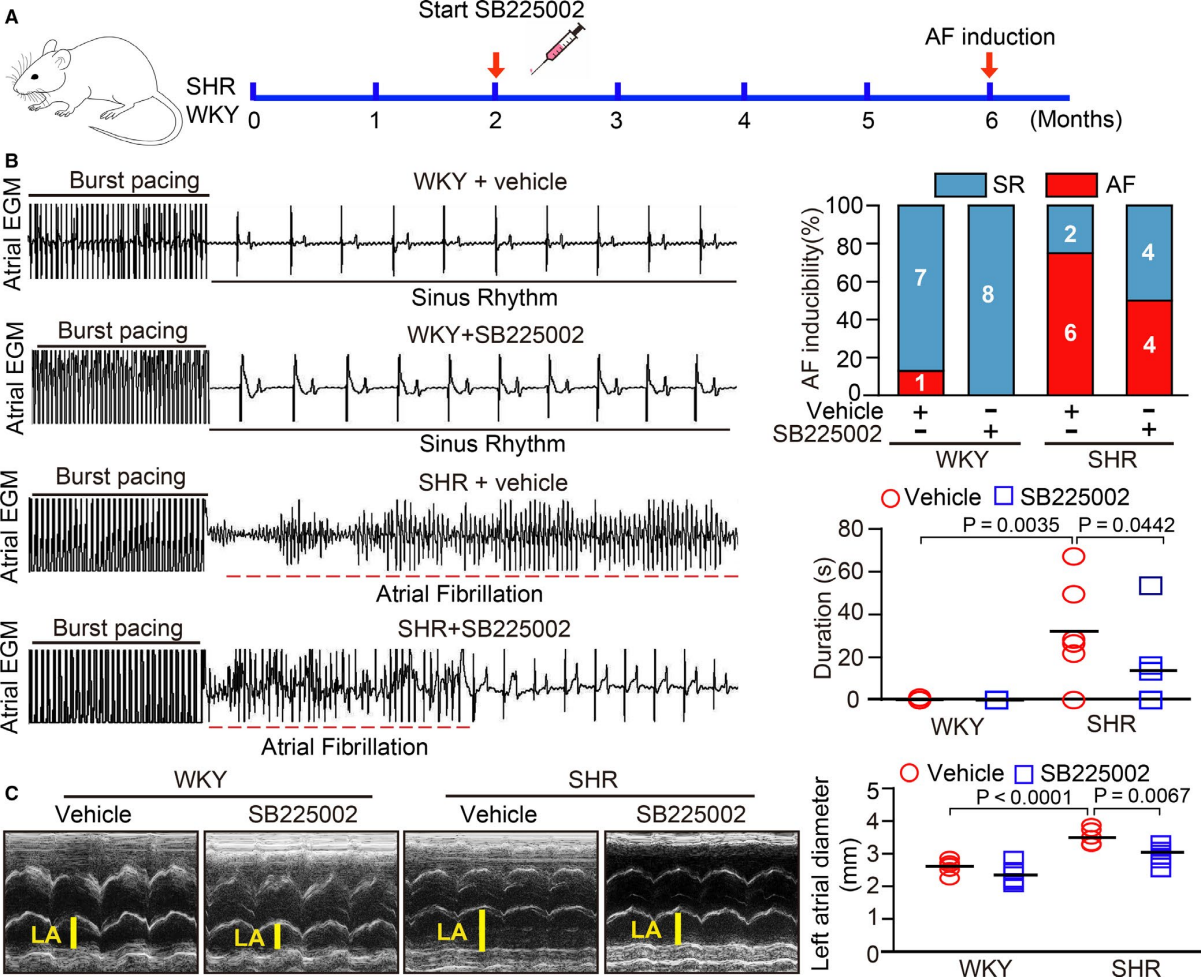
Pharmacological inhibition of CXCR2 by SB225002 prevents AF susceptibility, and left atrial dilation in SHRs. A, WKYs and SHRs at 2 mo of age were administered SB225002 intraperitoneally at a dose of 1 mg/kg/d for 4 mo. B, Representative atrial electrogram recordings (left). Solid underlines indicate burst pacing, and dashed underlines indicate AF. Percentage of successful AF induction (upper, n = 8). Duration of AF in rats after AF induction (lower). C, M‐mode echocardiography of the left atrial (LV) chamber (left). Quantification of LV diameter (right, n = 8). Results are expressed as the mean ± SEM, and n represents the number of animals in each group

SHRs reportedly show a mild elevation of blood pressure (140 mm Hg) at 2 months of age and compensatory cardiac hypertrophic remodelling.^14^ To evaluate whether CXCR2 is involved in the development of AF in SHRs, we started to treat 2‐month‐old WKYs and SHRs with the CXCR2 inhibitor SB225002 or vehicle and continued treatment for 4 months. In agreement with our previous study,^14^ systolic blood pressure (SBP) was highly elevated in SHRs compared to WKYs, and this was lowered in SB225002‐treated SHRs (Figure S1A). Moreover, 6‐month‐old SHRs showed a significant increase in inducibility (75% vs 12.5%), as reflected by an increased induction rate and duration of AF by electrical stimulation, compared with WKYs, whereas this effect was markedly attenuated in SB225002‐treated SHRs (50% vs 75%; Figure 2A). Accordingly, echocardiography indicated that the hypertension‐induced left atrial dilation observed in vehicle‐treated SHRs was also markedly blunted in SB225002‐treated SHRs (Figure 2B). Thus, these data demonstrate that CXCR2 is required for the development of AF in SHRs. There was no difference in these parameters between vehicle and SB225002 treatment in WKYs (Figure 2A,B). These data indicate that CXCR2 inhibition improves AF inducibility and atrial structural remodelling in SHRs.

### 3.3 SB225002 administration inhibits atrial fibrosis, macrophage infiltration and superoxide production

**FIGURE 3:**
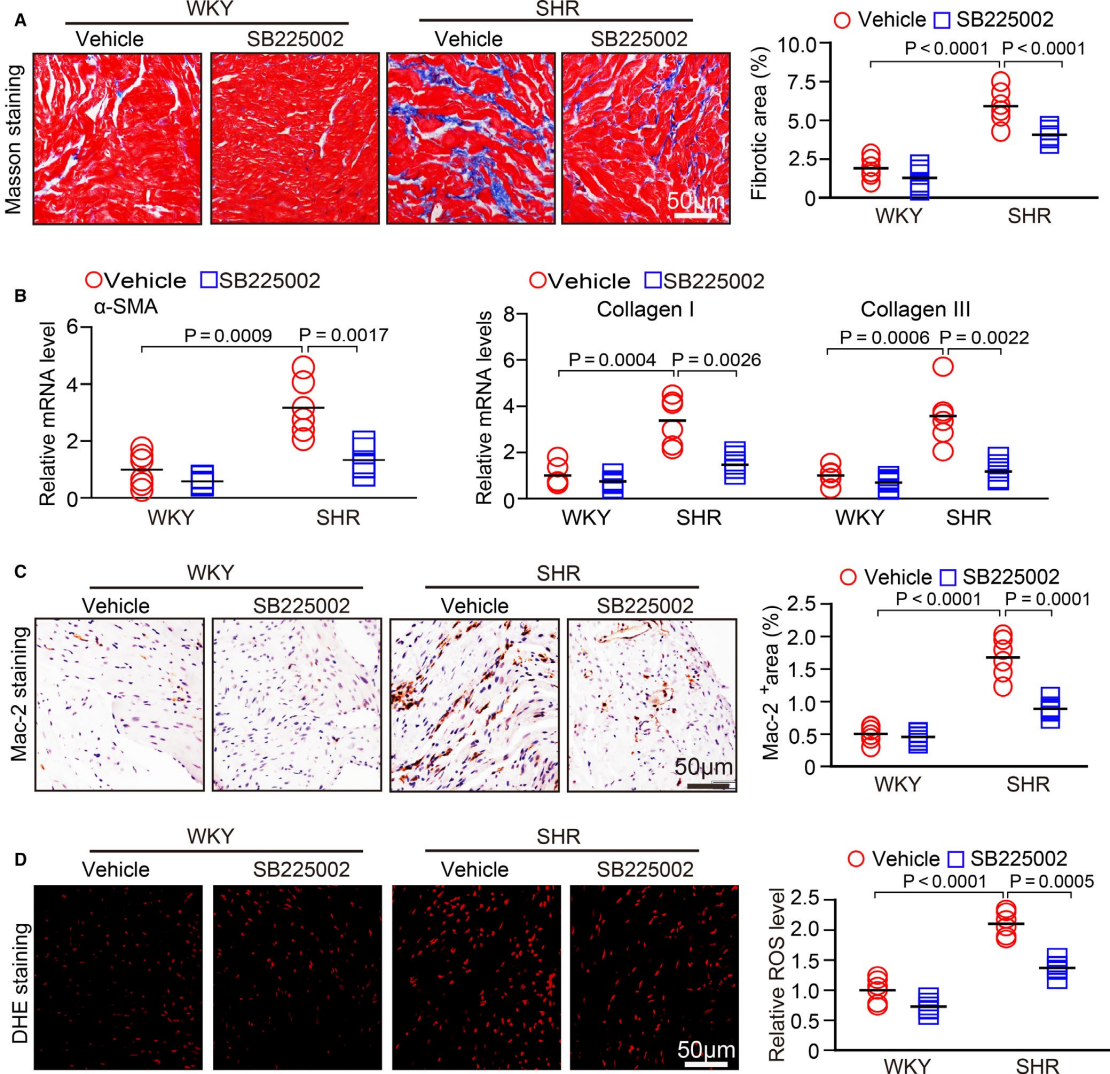
SB225002 administration prevents atrial fibrosis, macrophages infiltration and superoxide production in SHRs. A, WKYs and SHRs at 2 mo of age were administered SB225002 intraperitoneally at a dose of 1 mg/kg/d for 4 mo. Collagen deposition in left atrial sections detected by Masson's trichrome staining (left). Quantification of fibrotic areas (right, n = 6). B, qPCR analysis of the mRNA levels of α‐SMA, collagen I and collagen III in the atria from WKYs and SHRs (n = 6). C, Immunohistochemical staining of macrophages with an anti‐Mac‐2 antibody (left). Quantification of the Mac‐2^+^ area (right, n = 6). D, DHE staining of atrial superoxide production (left). Quantification of DHE intensity (right, n = 6). Scale bar: 50 μm. Results are expressed as the mean ± SEM, and n represents the number of animals in each group

As atrial fibrosis is the hallmark of structural remodelling in AF, we assessed whether CXCR2 affects the formation of atrial fibrosis. At 6 months of age, the atria of SHRs had a significantly increase of fibrotic area and the mRNA expression levels of α‐SMA, collagen I and collagen III compared with WKYs, but this increase was lower in SB225002‐treated SHRs (Figure 3A,B). Because CXCR2 mediates pro‐inflammatory macrophage recruitment and reactive oxygen species production in blood vessels and the heart,^12^, ^13^, ^14^ we assessed this effect in atrial tissue. As expected, SB225002 treatment markedly suppressed the atrial recruitment of Mac‐2^+^ macrophages and superoxide formation as indicated by DHE staining in comparison with vehicle treatment in SHRs (Figure 3C,D), suggesting that inhibiting CXCR2 prevents atrial macrophage recruitment, superoxide production and subsequent fibrosis.

### 3.4 SB225002 administration reverses pre‐established AF, atrial remodelling, inflammation and oxidative stress in SHRs

**FIGURE 4:**
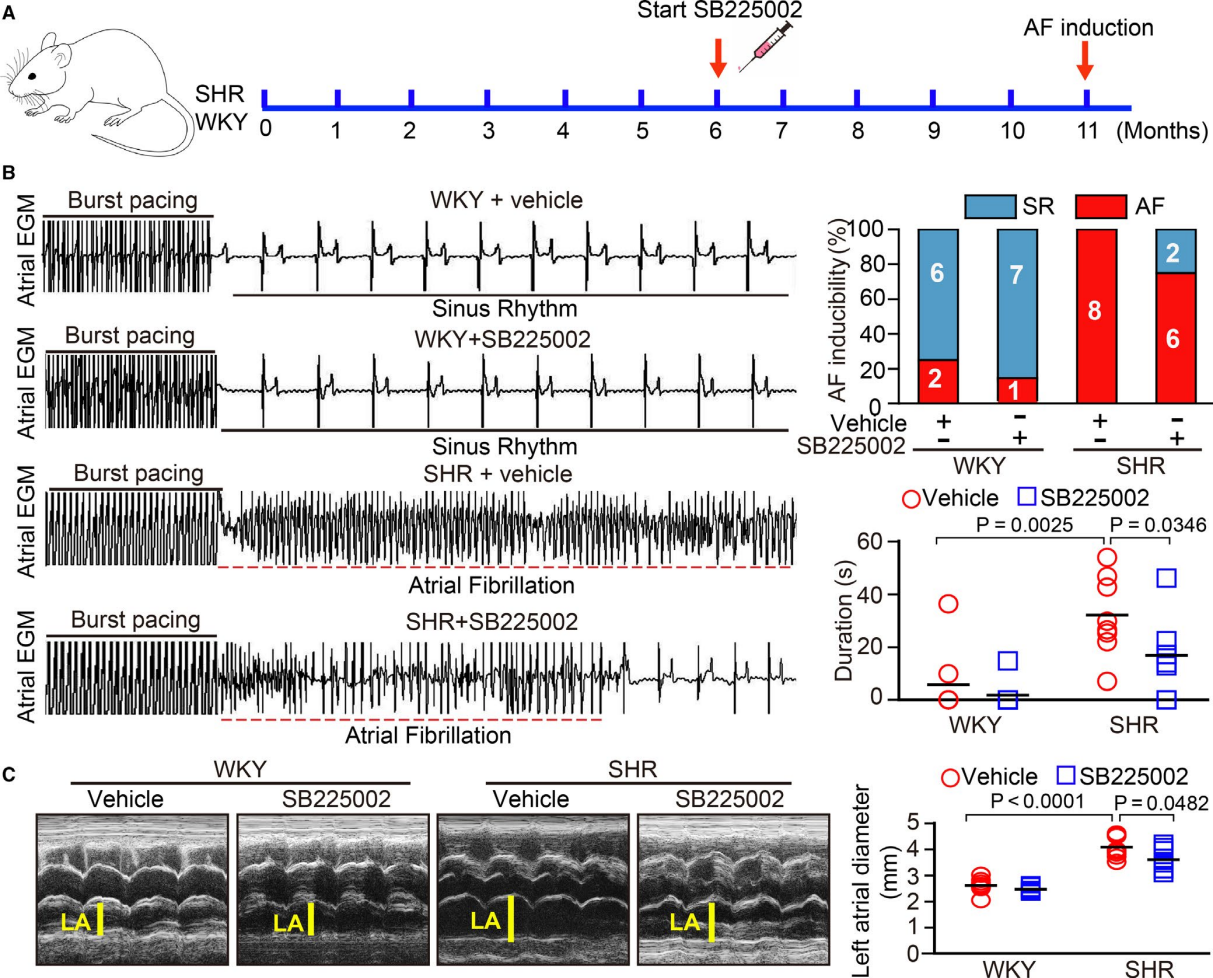
SB225002 administration reverses AF susceptibility and left atrial dilation in SHRs. A, WKYs and SHRs at 6 mo of age were administered the CXCR2 inhibitor SB225002 (1 mg/kg/d) or vehicle (castor oil) intraperitoneally for 5 mo. B, Representative atrial electrogram recordings for each group (left). Solid underlines indicate burst pacing, and dashed underlines indicate AF. Percentage of successful AF induction (upper, n = 8). Duration of AF in rats after AF induction (lower). C, Echocardiographic measurement of the left atrial (LV) chamber (left). Quantification of LV diameter (right, n = 8). Results are expressed as the mean ± SEM, and n represents number of animals in each group

**FIGURE 5:**
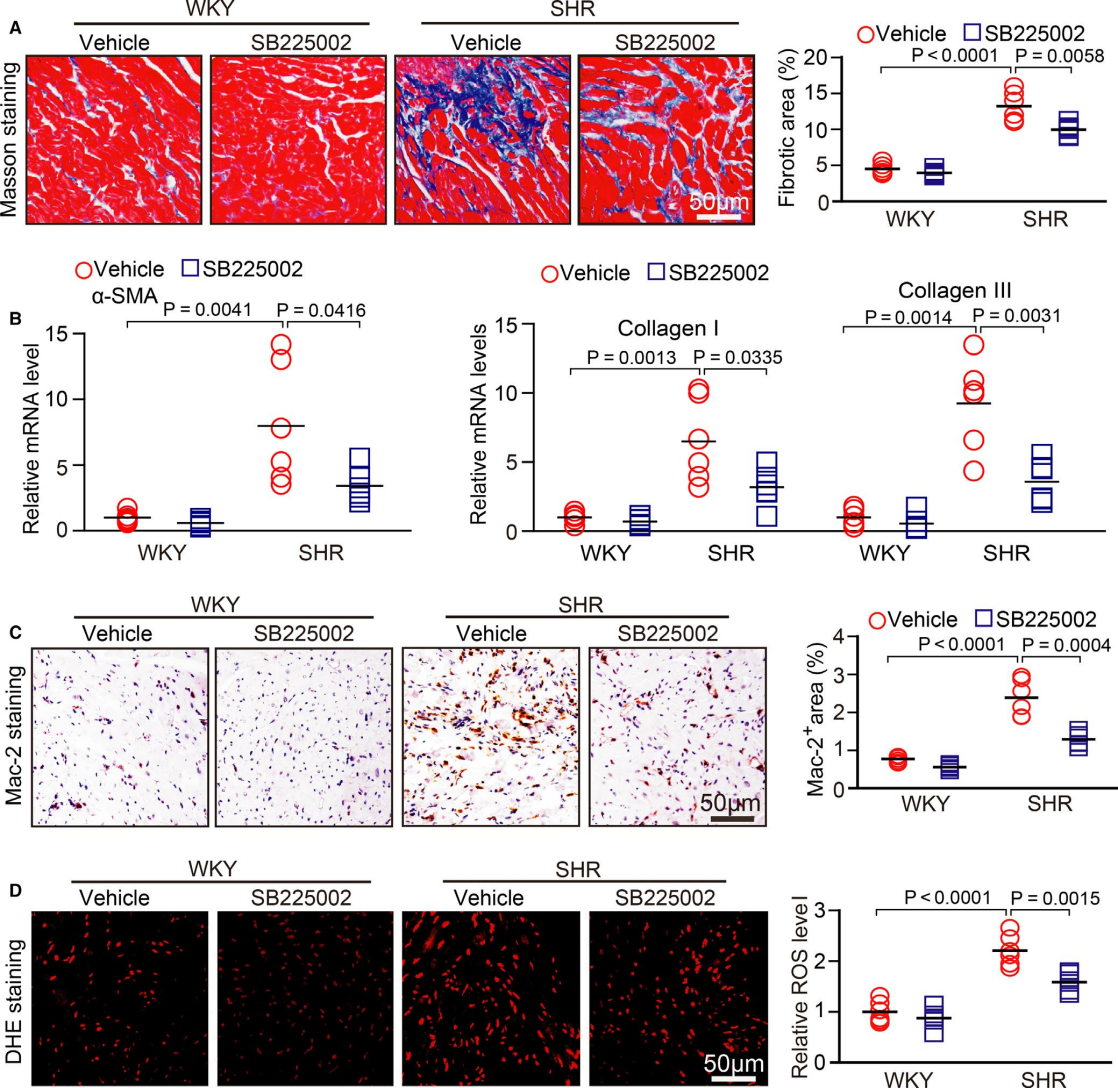
SB225002 administration prevents atrial fibrosis, macrophages infiltration and superoxide production in SHRs. A, WKYs and SHRs at 6 mo of age were administered SB225002 intraperitoneally at a dose of 1 mg/kg/d for 5 mo. Fibrosis of left atrial tissue was examined by Masson's trichrome staining (left). Quantification of fibrotic areas (right, n = 6). B, qPCR analysis of the mRNA levels of α‐SMA, collagen I and collagen III in the atria from WKYs and SHRs (n = 6). C, Immunohistochemical staining of macrophages with an anti‐Mac‐2 antibody (left). Quantification of the Mac‐2^+^ area (right, n = 6). D, DHE staining of atrial superoxide production (left). Quantification of DHE intensity (right, n = 6). Scale bar: 50 μm. Results are expressed as the mean ± SEM, and n represents number of animals in each group

We next tested whether inhibition of CXCR2 can reverse pre‐established atrial remodelling and AF in SHRs. At 6 months of age, WKYs or SHRs were randomly divided into two groups and intraperitoneally injected with the inhibitor SB225002 (1 mg/kg/d per rat) or vehicle for 5 months (Figure 4A). At 11 months of age, SHRs displayed a marked increase in SBP, inducibility and duration of AF, and left atrial diameter compared with WKYs, all of which were remarkably attenuated in SB225002‐treated SHRs (Figure 4B,C). Moreover, SB225002 administration also reduced atrial interstitial fibrosis, the mRNA expression levels of α‐SMA, collagen I, and collagen III, infiltration of Mac‐2^+^ macrophages and superoxide formation compared with vehicle‐treated control SHRs (Figure 5A‐D).

### 3.5 SB225002 inhibits the CXCR2‐mediated activation of downstream signalling pathways in SHRs

**FIGURE 6:**
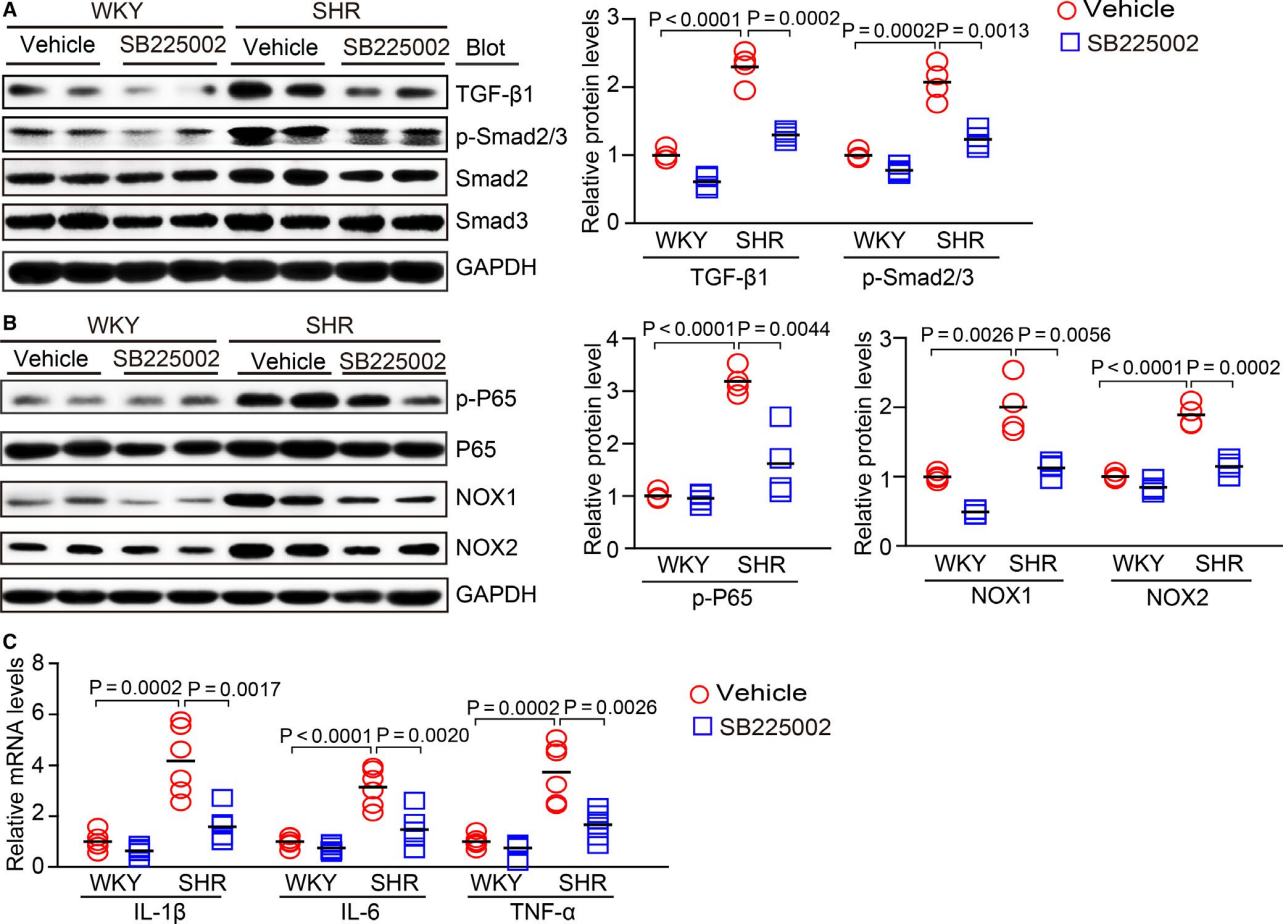
SB225002 treatment inhibits the activation of pro‐inflammatory, pro‐fibrotic and pro‐oxidative signalling pathways in the atria of SHRs. A, WKYs and SHRs at 2 mo of age were administered SB225002 intraperitoneally at a dose of 1 mg/kg/d for 4 mo. Immunoblotting analysis of TGF‐β1, p‐Smad2/3 and Smad2/3 protein levels in the atria (left). Quantification of each protein band (right, n = 4). B, Immunoblotting analysis of p‐P65, P65, NOX1 and NOX2 protein levels in the atria (left). Quantification of each protein band (right, n = 4). GAPDH was used as an internal control. C, qPCR analysis of the mRNA levels of IL‐1β, IL‐6 and TNF‐α in the atria from WKYs and SHRs (n = 6). Results are expressed as the mean ± SEM, and n represents number of animals in each group

To elucidate the precise mechanisms by which SB225002 inhibits AF development, we examined the downstream signalling mediators of CXCR2, TGF‐β1 and Smad2/3, which are central mediators of fibrogenesis. Indeed, TGF‐β1 and p‐Smad2/3 protein levels were significantly higher in the atria of vehicle‐treated SHRs than in control WKYs, whereas this effect was markedly attenuated in SB225002‐treated SHRs (Figure 6A). Moreover, we analysed the effect of SB225002 on the activation of NF‐κB signalling, a key regulator of pro‐inflammatory mediators and NADPH oxidase subunits. Similarly, the up‐regulation of p‐P65‐NF‐κB, NOX1 and NOX2 in the atria of saline‐treated SHRs was also effectively abrogated in SB225002‐treated SHRs (Figure 6B). In addition, the mRNA levels of pro‐inflammatory markers (IL‐1β, IL‐6 and TNF‐α) were lower in the atria of SB225002‐treated SHRs compared with the vehicle‐treated SHRs (Figure 6C,D).

### 3.6 SB225002 improves the abnormal electrical activity of atrial myocytes isolated from SHRs

**FIGURE 7:**
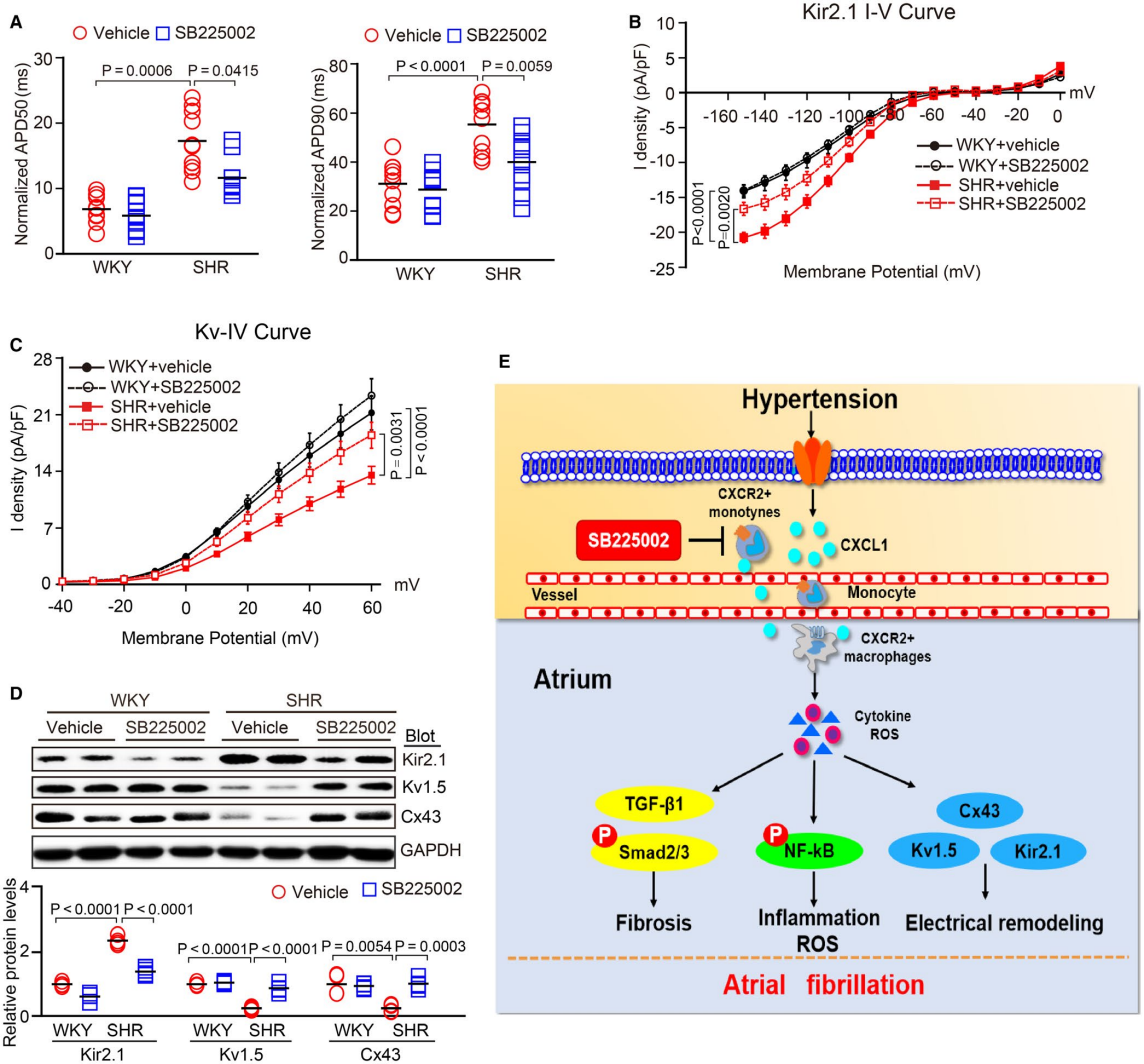
SB225002 improves atrial conduction abnormalities in atrial myocytes from SHRs. A, WKYs and SHRs at 2 mo of age were administered SB225002 intraperitoneally at a dose of 1 mg/kg/d for 4 mo. Atrial myocytes were isolated from the left atria of 6‐mo‐old WKYs and SHRs. Measurement of APDs at 50% and 90% recovery to baseline (APD50 and APD90) (n = 10 cells). B, Mean Kir2.1 current density (pA/pF) to voltage (mV) relationship (I‐V) in atrial myocytes obtained from each group (n = 10 cells). C, Mean Kv current density (pA/pF) to voltage (mV) relationship (I‐V) in atrial myocytes (n = 10 cells). D, Immunoblotting analysis of Kir2.1, Kv1.5 and Cx43 protein levels in atria from 6‐month‐old SHRs and WKYs (upper) and quantification of each protein band (lower, n = 4). GAPDH was used as an internal control. n represents myocytes or number of animals in each group. Results are expressed as the mean ± SEM, and n represents number of samples in each group. E, A working model for CXCR2‐mediated AF development. Hypertension up‐regulates CXCL1 expression to recruit CXCR2^+^ macrophages into the atrium, which trigger inflammation, oxidative stress to induce atrial fibrosis and electrical abnormalities, thereby leading to AF in SHRs. In contrast, pharmacological blockage of CXCR2 with SB225002 blunted these effects

We further examined whether CXCR2 inhibition regulates atrial electrophysiology. The action potential duration (APD) at 50% and 90% repolarization was recorded in atrial myocytes from 6‐month‐old SHRs and WKYs. APD50 and APD90 were significantly longer in SHR myocytes than in WKY control myocytes, and this effect was markedly reduced in SHR myocytes after 4 months of SB225002 treatment (Figure 7A). Because inward‐rectifier K^+^ (Kir) channels and voltage‐gated K^+^ (Kv) channels are considered to be important regulators of electrophysiology in AF,^18^ we measured the current density of Kir2.1 (a main member of the Kir2 subfamily) and Kv channels in isolated atrial myocytes. Recordings showed that Kir2.1 density was markedly higher, but Kv density was lower in SHR cells compared with WKY control cells, and this change was reversed in SB225002‐treated atrial myocytes (Figure 7B,C). Correspondingly, we verified the effect of CXCR2 inhibition on the protein levels of Kir2.1 and Kv1.5 (a member of the Kv subfamily) in the atria of WKYs and SHRs. The down‐regulation of Kir2.1 protein and up‐regulation of Kv1.5 protein were observed in SB225002‐treated SHR cells compared with vehicle‐treated SHR cells (Figure 7D). The protein level of Cx43 was also markedly lower in SB225002‐treated SHRs compared with vehicle‐treated SHRs (Figure 7D). There was no significant difference in these parameters between SB225002‐ and the vehicle‐treated WKYs (Figure 7A‐D).

## 4 DISCUSSION

The results of this study showed that CXCR2 has an important role in promoting the atrial recruitment of macrophages and the development of AF in hypertensive rats. Specifically, CXCL1 and CXCR2 expression was significantly up‐regulated in the atria of SHRs. Pharmacological inhibition of CXCR2 using SB225002 prevented and even reversed hypertension‐induced AF susceptibility and atrial remodelling in SHRs, likely though the inhibition of macrophage infiltration and oxidative stress. Moreover, the administration of SB225002 also inhibited the activation of pro‐inflammatory, oxidative stress and pro‐fibrotic signalling pathways (NF‐κB‐P65, NOXs and TGF‐β1/Smad2/3) in the atria of SHRs and improved conduction abnormalities (decreased ADP50, APD90 and IK1 current, but increased Kv current) in atrial myocytes. A working model is illustrated in Figure 7E.

Monocytes and macrophages are closely associated with the pathogenesis of the inflammatory response and related cardiovascular diseases. The recruitment of these cells is a key step in the initiation and development of heart failure and AF.^19^ This process is mainly regulated by various chemokines and adhesion molecules.^20^ Recent studies have demonstrated that chemokine‐receptor pathways are involved in regulating monocyte and macrophage recruitment to the sites of injury in diverse cardiovascular diseases. The up‐regulation of CXCL1‐CXCR2 plays a critical role in mediating monocyte infiltration, pro‐inflammatory responses and oxidative stress, by stimulating a variety of cell signalling pathways, including NF‐κB, AKT/mTOR, ERK1/2, STAT3, NADPH oxidase and TGF‐β1/Smad2/3 thereby leading to hypertension and cardiac remodelling in different animal models.^12^, ^13^, ^14^ However, whether CXCR2 mediates the infiltration of monocytes and the initiation of AF secondary to hypertension is unclear. Here, our results showed that CXCL1 and CXCR2 expression was highly up‐regulated in the atria of SHRs (Figure 1). In contrast, pharmacological inhibition of CXCR2 markedly reduced and reversed the atrial infiltration of Mac‐2^＋^‐macrophages, superoxide production, fibrosis and AF inducibility in SHRs (Figures 2, 3, 4, 5). Thus, this study further demonstrates that CXCR2 plays a role in the development of AF in hypertensive rats. Selectively targeting CXCR2 may be a novel strategy for the prevention and treatment of AF.

Atrial fibrosis is a hallmark of the structural remodelling in AF. The activation of TGF‐β1/Smad2/3 signalling is essential for the induction of atrial fibrosis, which may be sufficient to increase susceptibility to AF.^5^, ^6^ Experimental data have shown a link between inflammation and atrial fibrosis. For example, atrial leucocyte infiltration and pro‐inflammatory cytokines, such as TNF‐α and CRP, are increased in patients chronic AF or with non‐valvular AF.^21^, ^22^ Moreover, the infiltration of macrophages, release of pro‐inflammatory cytokines (IL‐1, IL‐6, TNF‐α, and MCP‐1) and superoxide levels are up‐regulated in Ang II‐induced mouse AF models, which may promote the development of fibrosis and the related structural and electrical remodelling of AF.^5^, ^6^ In the present study, we found that hypertension stimulated CXCR2 activation, which activated several downstream signalling pathways, including NF‐κB, NADPH oxidase and TGF‐β1/Smad2/3, which promoted pro‐inflammatory, pro‐oxidative and pro‐fibrotic effects that led to AF. In contrast, blocking CXCR2 markedly prevented and reduced these deleterious effects (Figure 6). Thus, our results support the notion that CXCR2‐mediated inflammation, oxidative stress and atrial fibrosis are involved in the pathogenesis of AF.

Atrial electrical remodelling is mediated predominantly by alterations to ion channels, which are also regulated by Ang II, inflammation, reactive oxygen species and fibrosis.^1^, ^23^, ^24^ Potassium channels, such as Kir2.1 and Kv1.5, play key roles in the cellular electrophysiology of AF, including resting membrane potential and APD.^18^ Kir2.1 is encoded by KCNJ2 for the inward‐rectifier K^+^ current(IK1). Kv1.5 is encoded by KCNA5 for the transient outward current (Ito).^18^ Interestingly, increased Kir2.1 expression and IK1 are observed in animals and patients with AF, and are critically involved in AF‐related atrial electrical remodelling.^25^, ^26^ Furthermore, the expression of Kv1.5 and Kv channels is reduced in chronic AF and is associated with prolonged APDs and AF.^26^, ^27^ In agreement with these reports, a whole‐cell patch‐clamp assay revealed that SB225002 markedly reduced ADP50, APD90 and the IK1 current, but increased the Kv current compared with vehicle in atrial myocytes from SHRs (Figure 7A‐C). Consistently, immunoblotting showed that the up‐regulation of Kir2.1 expression and down‐regulation of Kv1.5 and Cx43 expression in the atria of vehicle‐treated SHRs were markedly reversed in the atria of SB225002‐treated SHRs (Figure 7E), demonstrating a role for CXCR2 in regulating electrophysiological properties. However, whether CXCR2 is involved in other ion channels remains to be elucidated.

In conclusion, this study identified an important functional role of CXCR2 in promoting macrophage recruitment into the atria of SHRs, leading to atrial remodelling and AF inducibility after hypertension. Pharmacological blockage of CXCR2 by SB225002 significantly prevented and suppressed the development of AF, likely by inhibiting the hypertension‐induced recruitment of macrophages into the atria and oxidative stress in SHRs. Selective blocking of CXCR2 may represent a new approach for treating hypertensive AF. Further studies are needed to confirm effects of CXCR2 on AF in knockout mice and Ang II‐induced animal models and to elucidate how CXCR2 triggers atrial fibrosis and ion channels in vivo and in vitro.

## CONFLICT OF INTEREST

The authors declare no potential conflicts of the interest.

## AUTHOR CONTRIBUTION

**Hui‐Hua Li:** Writing‐original draft (lead). **Yun‐Long Zhang:** Data curation (lead); Formal analysis (lead). **Fei Teng:** Data curation (equal). **Xiao Han:** Data curation (supporting). **Xiao Yan:** Data curation (equal). **Pang‐Bo Li:** Formal analysis (supporting). **Shu‐Bin Guo:** Writing‐review & editing (equal).

## Supporting information

Table S1

Figure S1

## Data Availability

The data that support the findings of this study are available from the corresponding author upon reasonable request.

## References

[1] Hu YF , Chen YJ , Lin YJ , et al. Inflammation and the pathogenesis of atrial fibrillation. Nat Rev Cardiol. 2015;12:230‐243. 25622848 10.1038/nrcardio.2015.2

[2] Guo Y , Lip GY , Apostolakis S . Inflammation in atrial fibrillation. J Am Coll Cardiol. 2012;60:2263‐2270. 23194937 10.1016/j.jacc.2012.04.063

[3] Frustaci A , Chimenti C , Bellocci F , et al. Histological substrate of atrial biopsies in patients with lone atrial fibrillation. Circulation. 1997;96:1180‐1184. 9286947 10.1161/01.cir.96.4.1180

[4] Yamashita T , Sekiguchi A , Iwasaki YK , et al. Recruitment of immune cells across atrial endocardium in human atrial fibrillation. Circ J. 2010;74:262‐270. 20009387 10.1253/circj.cj-09-0644

[5] Li J , Wang S , Bai J , et al. Novel role for the immunoproteasome subunit PSMB10 in angiotensin ii‐induced atrial fibrillation in mice. Hypertension. 2018;71:866‐876. 29507100 10.1161/HYPERTENSIONAHA.117.10390

[6] Li J , Wang S , Zhang YL , et al. Immunoproteasome subunit beta5i promotes Ang II (Angiotensin II)‐induced atrial fibrillation by targeting ATRAP (Ang II Type I Receptor‐Associated Protein) degradation in mice. Hypertension. 2019;73:92‐101. 30571551 10.1161/HYPERTENSIONAHA.118.11813

[7] Blanchet X , Langer M , Weber C , et al. Touch of chemokines. Front Immunol. 2012;3:175. 22807925 10.3389/fimmu.2012.00175 PMC3394994

[8] Tarzami ST , Miao W , Mani K , et al. Opposing effects mediated by the chemokine receptor CXCR2 on myocardial ischemia‐reperfusion injury: recruitment of potentially damaging neutrophils and direct myocardial protection. Circulation. 2003;108:2387‐2392. 14568904 10.1161/01.CIR.0000093192.72099.9A

[9] Ritzman AM , Hughes‐Hanks JM , Blaho VA , et al. The chemokine receptor CXCR2 ligand KC (CXCL1) mediates neutrophil recruitment and is critical for development of experimental Lyme arthritis and carditis. Infect Immun. 2010;78:4593‐4600. 20823213 10.1128/IAI.00798-10 PMC2976349

[10] Boisvert WA , Santiago R , Curtiss LK , et al. A leukocyte homologue of the IL‐8 receptor CXCR‐2 mediates the accumulation of macrophages in atherosclerotic lesions of LDL receptor‐deficient mice. J Clin Invest. 1998;101:353‐363. 9435307 10.1172/JCI1195 PMC508574

[11] Soehnlein O , Drechsler M , Doring Y , et al. Distinct functions of chemokine receptor axes in the atherogenic mobilization and recruitment of classical monocytes. EMBO Mol Med. 2013;5:471‐481. 23417922 10.1002/emmm.201201717 PMC3598085

[12] Wang L , Zhang YL , Lin QY , et al. CXCL1‐CXCR2 axis mediates angiotensin II‐induced cardiac hypertrophy and remodelling through regulation of monocyte infiltration. Eur Heart J. 2018;39:1818‐1831. 29514257 10.1093/eurheartj/ehy085

[13] Wang L , Zhao XC , Cui W , et al. Genetic and pharmacologic inhibition of the chemokine receptor CXCR2 prevents experimental hypertension and vascular dysfunction. Circulation. 2016;134:1353‐1368. 27678262 10.1161/CIRCULATIONAHA.115.020754 PMC5084654

[14] Zhang YL , Geng C , Yang J , et al. Chronic inhibition of chemokine receptor CXCR2 attenuates cardiac remodeling and dysfunction in spontaneously hypertensive rats. Biochim Biophys Acta Mol Basis Dis. 2019;1865:165551. 31494226 10.1016/j.bbadis.2019.165551

[15] Zhang Y , Dedkov EI , Teplitsky D , et al. Both hypothyroidism and hyperthyroidism increase atrial fibrillation inducibility in rats. Circ Arrhythm Electrophysiol. 2013;6:952‐959. 24036190 10.1161/CIRCEP.113.000502 PMC3973490

[16] Parikh A , Patel D , McTiernan CF , et al. Relaxin suppresses atrial fibrillation by reversing fibrosis and myocyte hypertrophy and increasing conduction velocity and sodium current in spontaneously hypertensive rat hearts. Circ Res. 2013;113:313‐321. 23748429 10.1161/CIRCRESAHA.113.301646 PMC3774019

[17] Cao HJ , Fang J , Zhang YL , et al. Genetic ablation and pharmacological inhibition of immunosubunit beta5i attenuates cardiac remodeling in deoxycorticosterone‐acetate (DOCA)‐salt hypertensive mice. J Mol Cell Cardiol. 2019;137:34‐45. 31629736 10.1016/j.yjmcc.2019.09.010

[18] Luo X , Pan Z , Shan H , et al. MicroRNA‐26 governs profibrillatory inward‐rectifier potassium current changes in atrial fibrillation. J Clin Invest. 2013;123:1939‐1951. 23543060 10.1172/JCI62185 PMC3635715

[19] Shahid F , Lip GYH , Shantsila E . Role of monocytes in heart failure and atrial fibrillation. J Am Heart Assoc. 2018;7:e007849. 29419389 10.1161/JAHA.117.007849 PMC5850261

[20] Ha H , Debnath B , Neamati N . Role of the CXCL8‐CXCR1/2 axis in cancer and inflammatory diseases. Theranostics. 2017;7:1543‐1588. 28529637 10.7150/thno.15625 PMC5436513

[21] Ren M , Li X , Hao L , et al. Role of tumor necrosis factor alpha in the pathogenesis of atrial fibrillation: a novel potential therapeutic target? Ann Med. 2015;47:316‐324. 25982799 10.3109/07853890.2015.1042030

[22] Boldt A , Wetzel U , Lauschke J , et al. Fibrosis in left atrial tissue of patients with atrial fibrillation with and without underlying mitral valve disease. Heart. 2004;90:400‐405. 15020515 10.1136/hrt.2003.015347 PMC1768173

[23] Purohit A , Rokita AG , Guan X , et al. Oxidized Ca(2+)/calmodulin‐dependent protein kinase II triggers atrial fibrillation. Circulation. 2013;128:1748‐1757. 24030498 10.1161/CIRCULATIONAHA.113.003313 PMC3876034

[24] Xu J , Cui G , Esmailian F , et al. Atrial extracellular matrix remodeling and the maintenance of atrial fibrillation. Circulation. 2004;109:363‐368. 14732752 10.1161/01.CIR.0000109495.02213.52

[25] Melnyk P , Zhang L , Shrier A , et al. Differential distribution of Kir2.1 and Kir2.3 subunits in canine atrium and ventricle. Am J Physiol Heart Circ Physiol. 2002;283:H1123‐H1133. 12181143 10.1152/ajpheart.00934.2001

[26] Wang Z , Feng J , Shi H , et al. Potential molecular basis of different physiological properties of the transient outward K+ current in rabbit and human atrial myocytes. Circ Res. 1999;84:551‐561. 10082477 10.1161/01.res.84.5.551

[27] Brandt MC , Priebe L , Bohle T , et al. The ultrarapid and the transient outward K(+) current in human atrial fibrillation. Their possible role in postoperative atrial fibrillation. J Mol Cell Cardiol. 2000;32:1885‐1896. 11013132 10.1006/jmcc.2000.1221

