# Supplementary material for: Selective blocking of CXCR2 prevents and reverses atrial fibrillation in spontaneously hypertensive rats
Source: J Cell Mol Med. 2020 Aug 18;24(19):11272–82. doi: 10.1111/jcmm.15694 (PMC7576251; doi:10.1111/jcmm.15694)
Supplement: Supplementary file 1 — Table S1 [file JCMM-24-11272-s002.docx]

**Supplemental data**

**Table S1** Primers used in rat for quantitative real-time PCR analysis

| Gene | Forward Primer (5’- 3’) | Reverse Primer (5’- 3’) |
| --- | --- | --- |
| CXCR2 | TCGTAGAGCTACAGCAGGATTA | TTCTGGCGTTCACAGGTCTC |
| CXCL1 | TGCTAAAGGGTGTCCCCAAG | ACTCTCATCTCTCCGCCCTT |
| IL-1β | CTCTGTGACTCGTGGGATGATG | CCACTTGTTGGCTTATGTTCTGTC |
| IL-6 | TCTGCTCTGGTCTTCTGGAG | TTGCTCTGAATGACTCTGGC |
| TNF-α | TGATCGGTCCCAACAAGGA | TGCTTGGTGGTTTGCTACGA |
| Collagen I | GAGTACTGGATCGACCCTAACCA | GACGGCTGAGTAGGGAACACA |
| Collagen III | TCCCCTGGAATCTGTGAATC | TGAGTCGAATTGGGGAGAAT |
| α-SMA | TTCGTGACTACTGCTGAGCG | CTGTCAGCAATGCCTGGGTA |
| GAPDH | AGTGCCAGCCTCGTCTCATA | GATGGTGATGGGTTTCCCGT |

CXCR2, chemokine (C-X-C motif) receptor 2; CXCL1, Chemokine (C-X-C motif) ligand 1; IL-1β, Interleukin-1β; IL-6, Interleukin-6; TNF-α, Tumor necrosis factor; α-SMA, α-smooth muscle actin; GAPDH, Glyceraldehyde 3-phosphate dehydrogenase.

**Supplementary figure legends:**

**Figure 1. Administration of SB225002 prevents and reverses the elevation of blood pressure in hypertensive rats.**

1. WKYs and SHRs at 2 months of age were injected intraperitoneally with the CXCR2 inhibitor SB225002 (1 mg/kg/day) or vehicle (castor oil) for 4 months. SBP was measured every month by the tail-cuff method (n = 8). (**B**) WKYs and SHRs at 6 months of age were administered the CXCR2 inhibitor SB225002 (1 mg/kg/day) or vehicle (castor oil) intraperitoneally for 5 months. SBP was measured every month by the tail-cuff method (n = 8). Data are presented as the mean ± SEM, and n represents the number of animals in each group.
