# Supplementary figures and images for: Selective blocking of CXCR2 prevents and reverses atrial fibrillation in spontaneously hypertensive rats
Source: J Cell Mol Med. 2020 Aug 18;24(19):11272–82. doi: 10.1111/jcmm.15694 (PMC7576251; doi:10.1111/jcmm.15694)

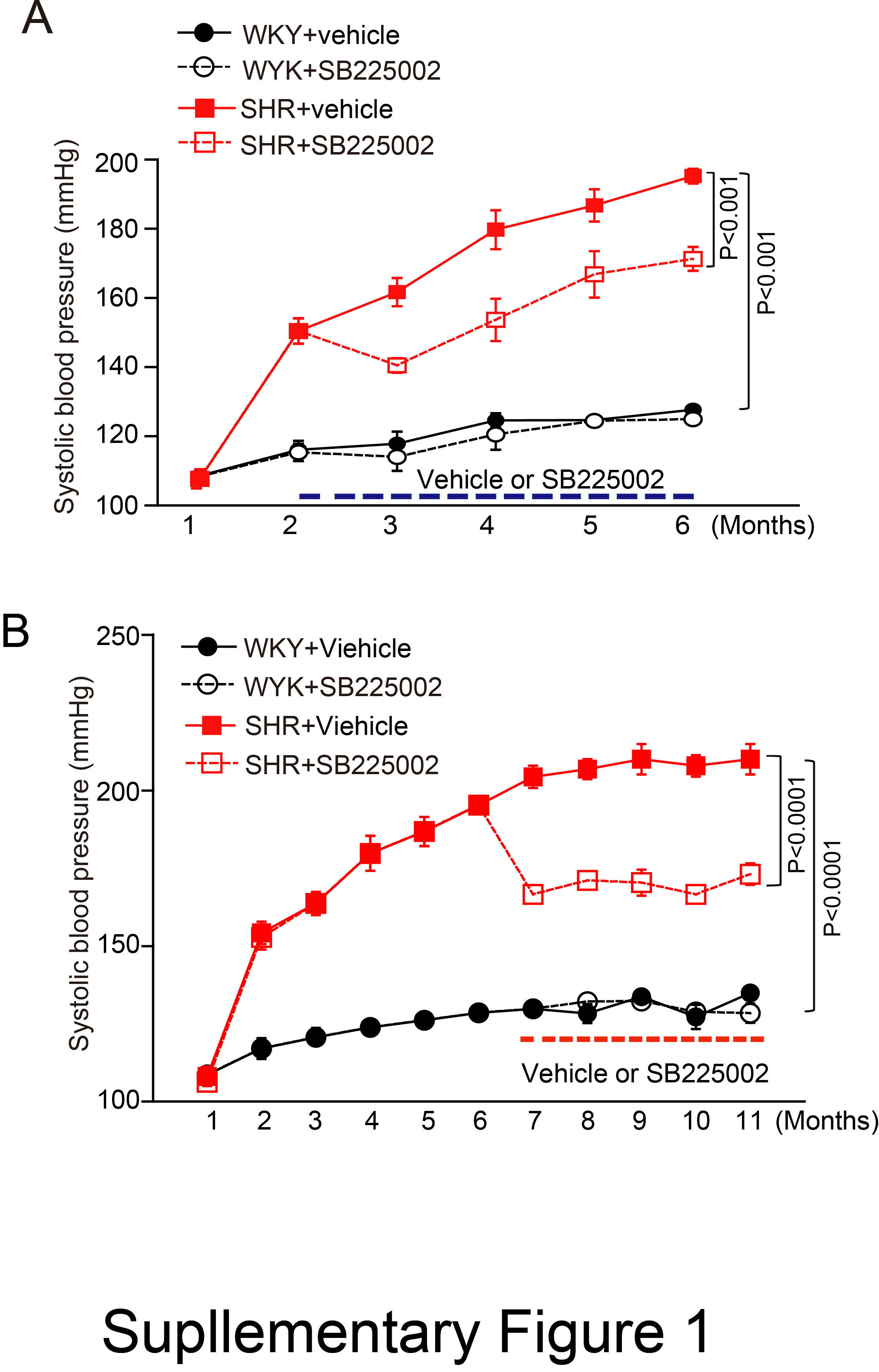

Supplement: Supplementary file 2 — Figure S1 [file JCMM-24-11272-s001.tif]
